# Injectable Hydrogel-Encapsulating Pickering Emulsion for Overcoming Lenvatinib-Resistant Hepatocellular Carcinoma via Cuproptosis Induction and Stemness Inhibition

**DOI:** 10.3390/polym16172418

**Published:** 2024-08-26

**Authors:** Xin Li, Chuanyu Tang, Hanjie Ye, Chihua Fang

**Affiliations:** 1 Department of Hepatobiliary Surgery I, General Surgery Center, Zhujiang Hospital, Southern Medical University, Guangzhou 510280, China; lixinlucky1314@163.com (X.L.); chuanyutang1932@163.com (C.T.); yehanjie1978@163.com (H.Y.); 2 Institute of Digital Intelligent Minimally Invasive Surger, Zhujiang Hospital, Southern Medical University, Guangzhou 510280, China; 3 Guangdong Provincial Clinical and Engineering Center of Digital Medicine, Guangzhou 510280, China; 4 South China Institute of National Engineering Research Center of Innovation and Application of Minimally Invasive Instruments, Guangzhou 510280, China

**Keywords:** hepatocellular carcinoma, lenvatinib resistant, cancer stemness, cuproptosis, hydrogel

## Abstract

Lenvatinib resistance (LenR) presents a significant challenge in hepatocellular carcinoma (HCC) treatment, leading to high cancer-related mortality rates globally. Unlike traditional chemotherapy resistance mechanisms, LenR in HCC is primarily driven by increased cancer cell stemness. Disulfiram, (DSF), functioning as a Cu ionophore, can coordinate with Cu^2+^ to overcome LenR in HCC by inhibiting cancer cell stemness and cuproptosis. However, DSF faces challenges due to its poor water solubility, while copper ions present issues related to systemic toxicity during widespread use. To address this, DSF and CuO nanoparticles (NPs) were co-encapsulated to form an oil-in-water Pickering emulsion (DSF@CuO), effectively elevating DSF and copper ion concentrations within the tumor microenvironment (TME). DSF@CuO was then combined with sodium alginate (SA) to form a DSF@CuO-SA solution, which gelatinizes in situ with Ca^2+^ in the TME to form a DSF@CuO Gel, enhancing Pickering emulsion stability and sustaining DSF and copper ion release. A DSF@CuO Gel exhibits enhanced stability and therapeutic efficacy compared to conventional administration methods. It effectively induces mitochondrial dysfunction and cuproptosis in LenR HCC cells by downregulating DLAT, LIAS, and CDKN2A, while upregulating FDX1. Furthermore, it suppresses cancer stemness pathways through activation of the JNK/p38 MAPK pathway and inhibition of the NF-κB and NOTCH signaling pathways. These findings suggest that DSF@CuO Gels are a promising therapeutic strategy for treating LenR HCC. In vivo and in vitro LenR HCC models demonstrated significant therapeutic efficacy. In conclusion, this novel approach underscores DSF@CuO Gel’s potential to overcome LenR in HCC, offering a novel approach to address this clinical challenge.

## 1. Introduction

Hepatocellular carcinoma (HCC) ranks among the deadliest cancers globally, placing fifth in cancer-related mortality for men and seventh for women, posing a significant threat to global health [1]. Due to its insidious onset, most patients with HCC are diagnosed in advanced stages, leading to the loss of surgical treatment opportunities for the majority of patients [2]. Lenvatinib, a multi-target kinase inhibitor, has shown superior progression-free survival (PFS) rates compared to sorafenib in a randomized, open-label phase III clinical trial involving 1492 HCC patients, leading to its approval as a first-line therapy for advanced HCC in 2018 [3,4]. However, the emergence of lenvatinib resistance (LenR) within months of treatment initiation has become a notable challenge, compromising therapeutic efficacy and patient survival [5]. Current research on LenR is limited, and its mechanisms are largely distinct from traditional chemotherapy involving P-glycoprotein (P-gp) activity [6]. Instead, the mechanism of LenR is primarily associated with the emergence of cancer stemness, which confers significant resistance to conventional strategies targeting apoptosis and cell proliferation inhibition [7,8,9]. Moreover, cancer stemness plays a pivotal role in HCC progression, tumor recurrence, and metastasis [10]. For instance, the hyaluronic acid receptor CD44, indicative of cancer stemness in HCC, correlates with increased cell adhesion, invasion, metastasis, and resistance to treatment [11]. Epithelial–mesenchymal transition (EMT) is closely associated with the invasion and metastasis of tumor cells and also signifies enhanced cancer stemness [12]. Thus, targeting cancer stemness and epithelial–mesenchymal transition (EMT)-related pathways emerges as a promising therapeutic approach for addressing LenR in HCC.

On the other hand, disulfiram (DSF), a drug employed for over six decades in treating alcohol dependence, exhibits well-established pharmacokinetics, safety, and tolerance at the US Food and Drug Administration (FDA)-recommended dosage [13]. Recently, DSF has gained attention for its potential to combat drug resistance in tumor treatment [14,15]. Furthermore, recent studies have reported that DSF can act as Cu ionophores, effectively transporting Cu^2+^ into tumor cells [16]. DSF can also react with Cu^2+^ to generate Cu^+^, causing mitochondrial oxidative phosphorylation dysfunction and subsequently inducing tumor cell cuproptosis [14,17]. Compared to other copper ion carriers, DSF not only transports copper ions into tumor cells to induce cuproptosis but also inhibits tumor cell stemness, thereby enhancing the overall therapeutic efficacy against tumors [18]. Emerging studies suggest that tumor cells acquire stem-like characteristics primarily through metabolic reprogramming, relying heavily on oxidative phosphorylation rather than glycolysis [19,20]. This suggests that DSF can address LenR HCC by inhibiting cancer cell stemness and cuproptosis. However, a recent study has indicated that copper ion levels are decreased in HCC tumor tissue while serum copper ions are elevated [21]. Despite DSF acting as a potent copper ion transporter, the low copper ion concentration in the tumor microenvironment (TME) makes it difficult to induce cuproptosis. Currently, most clinical trials and extensive animal experiments involving DSF and copper ions for cancer treatment have ended in failure [22]. The primary reasons include the poor water solubility of DSF, which reduces its activity after metabolism in the gastrointestinal tract, blood, and liver, thereby limiting its clinical utility [23,24,25]. Additionally, the human body’s limited tolerance for copper ions makes it challenging to achieve therapeutically effective concentrations at the tumor site through oral or intravenous administration [26], further contributing to the failure of these studies. Therefore, changing the traditional intravenous and oral administration strategies to in situ injection into the TME, employing responsive release and sustained release strategies, may offer a new solution to address the abovementioned issues.

In this regard, an emulsion is a multiphase system composed of two immiscible liquids, with one liquid dispersed in another [27]. Pickering emulsion is a novel system using solid nanoparticles as stabilizers to form emulsions [28]. Utilizing copper-containing nanoparticles as stabilizers can reduce systemic toxicity and achieve sustained release of copper ions in response to the acidic TME [29,30]. Moreover, adjusting the water-to-oil ratio can regulate the dosage of DSF administered during treatment. However, the stability of Pickering emulsion is significantly compromised in acidic microenvironments, leading to a considerable decrease in therapeutic efficacy. Lately, injectable hydrogels have emerged to gel in situ within the TME, significantly improving drug sustained release and therapeutic efficacy [31]. Encapsulating Pickering emulsion within injectable hydrogels in situ within the tumor microenvironment addresses these stability issues under acidic conditions, significantly enhancing the sustained release of DSF and copper ions, thereby improving therapeutic efficacy.

**Scheme 1:**
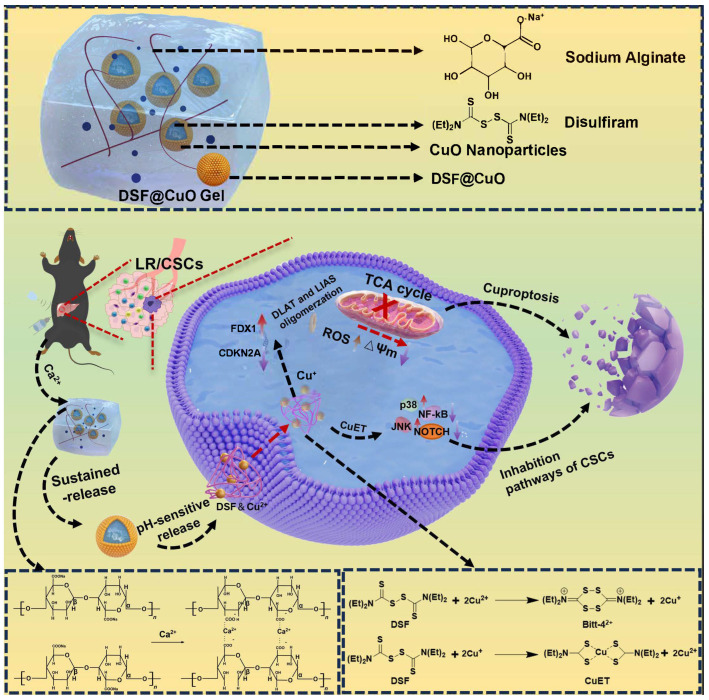
Schematic illustration of injection of DSF@CuO Gel hydrogel for treatment of LenR HCC.

In summary, this study developed a DSF@CuO Gel, an innovative injectable in situ hydrogel aimed at targeting and eliminating LenR HCC cells. This formulation significantly enhances the sustained release of DSF and Cu^2+^ within the low pH TME. DSF and CuO nanoparticles were incorporated into a complex aqueous–oil phase system under ultrasonic conditions, forming a Pickering emulsion (DSF@CuO). This emulsion was mixed with a sodium alginate (SA) solution to create DSF@CuO-SA. Upon injection into the HCC TME, the system cross-links with high concentrations of Ca^2+^, forming in situ a DSF@CuO Gel hydrogel, significantly enhancing stability and facilitating sustained release. DSF@CuO Gel induces mitochondrial dysfunction and cuproptosis in LenR HCC cells by downregulating DLAT, LIAS, and CDKN2A while upregulating FDX1. Furthermore, it suppresses LenR HCC cell stemness by activating the JNK/p38 MAPK pathway and inhibiting the NF-κB and NOTCH signaling pathways (Scheme 1). As a result, DSF@CuO Gel demonstrates promising therapeutic efficacy against LenR HCC both in vitro and in vivo, presenting a novel approach for treating LenR HCC.

## 2. Materials and Methods

### 2.1. Materials, Cell Lines, and Mice

CY7-NHS was procured from Xi’an Ruixi Biotechnology Co., Ltd., Xi’an, China, Cu(OAc)_2_·H_2_O, and Polyvinylpyrrolidone (PVP) was sourced from Ficus Discovery Platform Company Limited, Hainan, China. Corn oil and sodium alginate (SA) were acquired from MACKLIN. Human HCC cell lines Huh7 and Hep 3B, as well as the normal liver cell line LO-2, were obtained from Wuhan Pricella. After a nine-month period of gradually increasing lenvatinib concentration, this study successfully induced proliferation-normal Hep 3B and Huh7 cell lines under a lenvatinib concentration of 40 μM. The mouse HCC cell line Hepa-1-6-Luc was purchased from Wuhan Pricella. The HUVECs cell line was provided by Professor Jisheng Xiao’s research group. Huh7, Hepa-1-6, HUVECs, and LO-2 were cultured in Dulbecco’s Modified Eagle Medium containing 10% fetal bovine serum and 100 U/mL penicillin in an incubator set at 37 °C with 5% CO_2_. C57BL/6 mice and BALB/c rats aged 6–8 weeks were sourced from Zhuhai Bestest. All mouse studies were conducted following protocols approved by the Institutional Animal Care and Use Committee of Zhujiang Hospital, Southern Medical University.

### 2.2. Preparation and Characterization of CuO NPs

A total of 0.4 g of Cu(OAc)_2_·H_2_O was dissolved in 40 mL of ethanol solution and stirred until completely dissolved. Subsequently, 0.4 g of PVP was added, and the mixture was vigorously stirred to achieve a homogeneous solution. This solution was then transferred to a 50 mL hydrothermal reactor and subjected to a reaction at 110 °C for 20 h. Following the reaction, the reactor was allowed to naturally cool to room temperature. The resulting product was separated by centrifugation, washed three times with water and ethanol, and finally dried at 80 °C for storage.

### 2.3. Preparation and Characterization of DSF@CuO

CuO nanoparticles (NPs) were dispersed in pure water to form an aqueous phase. Subsequently, the squalene oil phase was ultrasonically mixed with the aqueous phase of CuO NPs, maintaining a water-to-oil ratio of 4:1 and a nanoparticle content of 0.4%, for a duration of 20 min. This was followed by probe sonication (scientz-IID, 1000 W, Ningbo Xinzhi Biotechnology Co., Ltd., Ningbo, China) for 15 min to generate DSF@CuO. The same method can be used to prepare Len@CuO.

### 2.4. Preparation and Characterization of DSF@CuO Gel

The sodium alginate (SA) was dissolved in water, and then the DSF@CuO solution was added. After thorough mixing, CaCl_2_ (100 mM) was introduced to investigate the gelation of the DSF@CuO Gel. Finally, we examined the ultrastructure of the DSF@CuO Gel hydrogel using a scanning electron microscope (SEM).

### 2.5. Rheological Characterization

The rheological properties of the hydrogels were examined using a rheometer (model AR-2000ex, TA Instruments, New Castle, DE, USA). Prior to the experiments, the gap between the rheometer plates was precisely set to 500 μm, and the temperature was maintained at a constant 37 °C to mimic physiological conditions. To evaluate the dynamic response of the hydrogels, a frequency sweep test was conducted at a fixed strain of 1% over a broad frequency range spanning from 0.1 to 100 rad/s. This allowed us to assess the elasticity (G′) and viscosity (G″) of the hydrogels under varying frequency conditions. Subsequently, a strain sweep test was performed to determine the strain crossover point of the hydrogels. This test was conducted at a constant frequency of 1 rad/s, with the strain varying from 0.1 to 1000%. By monitoring the transition from elastic (G′ > G″) to viscous (G′ < G″) behavior, we were able to characterize the strain response of the hydrogels and assess their potential for injectability and drug delivery applications.

### 2.6. Hemolysis Assay

This study initially involved the isolation of red blood cells from fresh mouse blood, which were then prepared into a red blood cell suspension (RCS) at a concentration of 2% *v*/*v*. Subsequently, the RCS suspension was incubated at 37 °C for 3 h with varying concentrations of DSF@CuO-SA solution (ranging from 15.125 to 500 μg/mL). Positive and negative control groups were established using RCS suspensions treated with deionized water and saline, respectively. Following centrifugation and photographic documentation, the supernatant from each solution was collected to measure UV-vis absorbance at 540 nm.

### 2.7. Differential Gene Selection and Gene Enrichment Analysis

In this study, we employed the limma package in R to analyze sequencing data obtained from experimental and control groups, aiming to identify differentially expressed genes (DEGs). The criteria for selecting DEGs were set at a significance level of *p* < 0.05, coupled with an absolute fold change in gene expression of ≥2. Utilizing the ggplot2 package in R, volcano plots of the DEGs were generated to visually depict both upregulated and downregulated genes. Subsequently, the lists of upregulated and downregulated genes were imported into Metascape (https://metascape.org) for further analysis. Within Metascape, enrichment analyses were conducted using default datasets for Gene Ontology (GO), Kyoto Encyclopedia of Genes and Genomes (KEGG), and other commonly used datasets. The objective was to elucidate the potential roles and significance of these DEGs in various biological processes, cellular components, molecular functions, and pathological pathways.

### 2.8. GSEA and GSVA Analysis

We performed Gene Set Enrichment Analysis (GSEA) on various datasets using GSEA software (version 4.3.2) with the following main parameters: permutation type set to “gene set”, and the number of permutations set to 1000. Subsequently, we utilized the ggplot2 package in R to generate GSEA plots, aiming to visually elucidate the changes in significantly altered pathways such as “NF-κB”. Additionally, we conducted Gene Set Variation Analysis (GSVA) on three datasets using the GSVA package in R (version 1.38). The gene sets employed in both GSEA and GSVA analyses were sourced from the Molecular Signatures Database (MSigDB, https://www.gsea-msigdb.org), encompassing the C2 curated gene sets, C5 ontology gene sets, and C7 immunologic signature gene sets. To further illustrate the changes in significantly altered pathways, we employed the ggplot2 package to generate bar charts.

### 2.9. Cytotoxicity Evaluation and IC50

The IC50 of the resistant strains to lenvatinib was determined using the CCK-8 assay, and the effects of various treatment groups on LenR strains were evaluated. Subsequently, a cell viability staining experiment was conducted to assess the cytotoxic effects of different treatment groups on 3D tumor spheroids. Corn oil (no additional treatment), DSF (0.8 μM final concentration), CuO NPs (0.8 μM final concentration), Len@CuO (40 μM final concentration and 0.8 μM CuO NP final concentrations), and DSF@CuO (0.8 μM DSF and 0.8 μM CuO NP final concentrations) were used for different treatment groups.

### 2.10. Inhibition of LenR HCC Cell Spheroid Formation Experiment

To prepare 50 mL of 3D spheroid culture medium, combine 1 mL of B27 supplement, 1 mL of bFGF (20 ng/mL), 1 mL of EGF (20 ng/mL), 0.2 g of BSA (0.4% concentration), and 25 μg of insulin (5 μg/mL concentration) in a suitable container. Adjust the total volume to 50 mL with an appropriate basal medium such as DMEM/F12 or RPMI-1640, ensuring thorough mixing. Divide the resulting solution into five equal portions, each containing 10 mL. Treat each portion according to the specified groups: corn oil (no additional treatment), DSF (0.8 μM final concentration), CuO NPs (0.8 μM final concentration), Len@CuO (40 μM final concentration and 0.8 μM CuO NP final concentrations), and DSF@CuO (0.8 μM DSF and 0.8 μM CuO NP final concentrations). Incubate the spheroids in their respective treatment media for 2 weeks. After the treatment period, transfer the spheroids onto microscope slides and capture photographs using a conventional optical microscope. Ensure adherence to sterile techniques and proper handling procedures to maintain cell viability and prevent contamination throughout the experiment.

### 2.11. Cell Cloning Experiment

Approximately 700 cells were seeded into each well of a 6-well plate to ensure an equal cell number across all groups. Once the cells adhered to the wells and attained stability, respective drugs were added to the different treatment groups. Throughout this period, the medium was refreshed every 3 days to maintain cell health. After a 14-day incubation period, the cells were fixed with 4% paraformaldehyde and subjected to crystal violet staining. Subsequently, photographs of each group were captured using a digital camera for further analysis.

### 2.12. Transwell Experiment

Matrix gel was applied to the bottom of each transwell upper chamber and incubated at 37 °C for 3 h to form a thin film. Following this, 100 μL of serum-free medium was added to each upper chamber and allowed to hydrate for 30 min in a culture incubator. In parallel, 500 μL of medium containing 5% FBS was added to the lower chamber, which was subsequently placed in a 24-well plate. Subsequently, 200 μL of cell suspension was added to each upper chamber, and the transwells were incubated in a 37 °C, 5% CO_2_ incubator for a duration of 12 to 48 h.

### 2.13. Western Blot for Cell Protein Expression

Cell proteins from various treatment groups were extracted and quantified using the BCA method to determine their concentrations. Subsequently, corresponding SDS-PAGE gels were prepared for electrophoresis, followed by the electrophoresis and membrane transfer processes to transfer the separated proteins onto a membrane. Afterward, the membrane was incubated with primary antibodies specific to the target proteins of interest, followed by incubation with secondary antibodies conjugated to an enzyme such as horseradish peroxidase (HRP). Finally, the detection of the target proteins was performed using methods such as chemiluminescence or chromogenic substrate, enabling the visualization of protein bands on the membrane. The antibodies used in this study were as follows: DLAT (proteintech, Human, 12 h), LIAS (proteintech, Human, 12 h), CDKN2A (proteintech, Human, 12 h), FDX1 (proteintech, Human, 12 h), Tubulin (affinity, Human, 12 h), EpCAM (affinity, Human, 12 h), SOX9 (affinity, Human, 12 h), CD24 (affinity, Human, 12 h), E-cadherin (affinity, Human, 12 h), N-cadherin (affinity, Human, 12 h), Vimentin (affinity, Human, 12 h), GADPH (affinity, Human, 12 h), p-p38 (affinity, Human, 12 h), JNK MAPK (affinity, Human, 12 h), p38 (affinity, Human, 12 h), p-JNK MAPK (affinity, Human, 12 h), NF-κB (affinity, Human, 12 h), p-NF-ΚB (affinity, Human, 12 h), NOTCH (affinity, Human, 12 h), and Goat Anti-Rabbit IgG (H+L) HRP (affinity, Rabbit, 1.5 h).

### 2.14. In Vivo Treatment of LenR BALB/c Nude Mice Model

When the resistant strains reached the logarithmic growth phase, they were harvested and implanted subcutaneously in nude mice. Subsequently, different treatment groups received therapy. Upon completion of the treatment regimen, mouse tumors were collected for photography to document any visible changes or responses to the treatments.

### 2.15. In Vivo Drug Retention Experiment

First, a subcutaneous tumor model in mice was induced using the Hepa 1-6 cell line. Once the tumor volume reached approximately 800–1000 mm^3^, three distinct groups were identified for in situ injection into the tumors: DSF and CY7-NHS, DSF@CuO, (containing CY7-NHS and DSF within the oil phase), and DSF@CuO Gel (also containing CY7-NHS and DSF within the oil phase). The retention of CY7-NHS in mice from the different groups was monitored using IVIS imaging.

### 2.16. Evaluation of In Situ HCC Model Treatment Efficacy

Hepa 1-6-luc cells were cultured as described previously in serum-free conditions to form spheroids. After collection, the spheroid-derived tumor cells were orthotopically injected into the livers of C57 mice to establish an orthotopic liver tumor model. The progression of liver tumors in mice was monitored using IVIS imaging.

### 2.17. Immunofluorescence Staining

Tumors were collected from mice and promptly frozen in an optimal cutting temperature compound. Using a cryostat, tumor sections were sliced, mounted on slides, and subjected to staining with different primary antibodies: DLAT (Proteintech, Wuhan, China, 13426-1-AP) and LIAS (Proteintech, Wuhan, China, 11577-1-AP) incubated overnight at 4 °C in accordance with the manufacturer’s instructions. Cy3 and FITC were utilized as secondary antibodies. Subsequently, slides were examined using a confocal microscope (Nikon, AX NIS-Elements, Tokyo, Japan). All antibodies used in the experiments were diluted 200 times.

### 2.18. In Vivo Biosafety Assessment in Mice

Following the completion of treatment, serum was collected from mice in the respective groups for the measurement of alanine transaminase (ALT), aspartate transaminase (AST), blood urea nitrogen (BUN), and creatinine (CR) levels to assess liver and kidney function. Additionally, major organs including the heart, liver, spleen, lungs, and kidneys were collected from mice in different treatment groups for hematoxylin and eosin (H&E) staining to detect histological changes.

### 2.19. Statistical Analysis

All data are presented as mean ± standard deviation. Statistical significance between groups was determined using a one-way ANOVA followed by Student’s *t*-test for pairwise comparisons. Statistical analyses were performed using GraphPad Prism 8.0, and a *p*-value of less than 0.05 was considered statistically significant (* *p* < 0.05, ** *p* < 0.01, and *** *p* < 0.001).

## 3. Results and Discussion

### 3.1. Preparation and Characterization of Injectable Hydrogel

**Figure 1:**
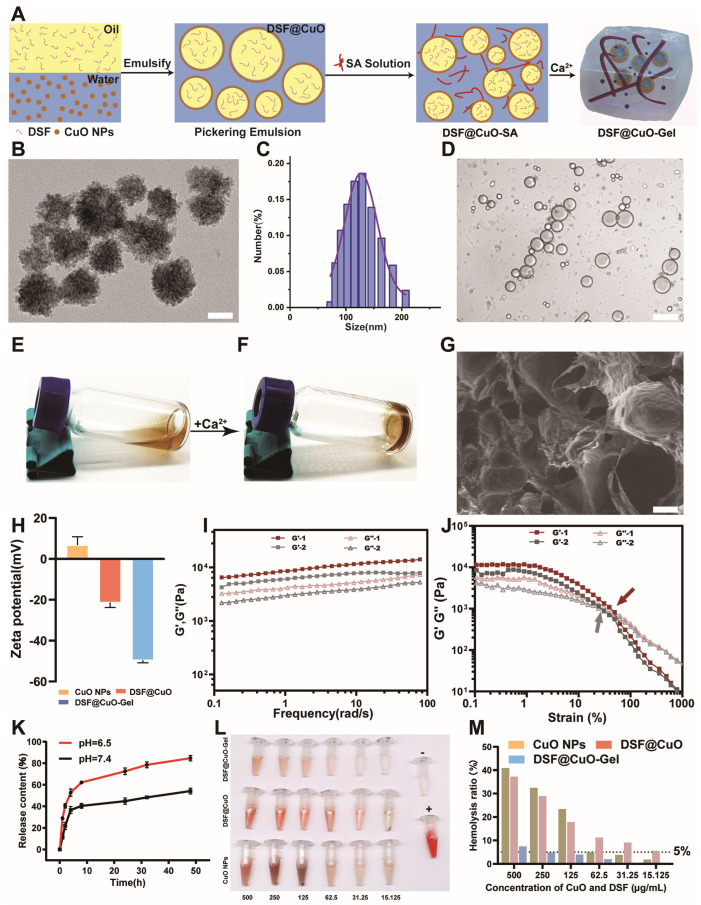
Characterization of injectable hydrogel DSF@CuO Gel. (**A**) Schematic diagram of DSF@CuO Gel synthesis. (**B**) TEM images of CuO NPs (Scale bar = 100 nm). (**C**) DLS was used to measure diameter of CuO NPs. (**D**) Images of DSF@CuO under confocal microscope (scale bar = 20 μm). (**E**,**F**) Photos of DSF@CuO Gel before and after addition of Ca^2+^. (**G**) SEM images of DSF@CuO Gel hydrogel (scale bar = 200 μm). (**H**) Zeta potential of CuO NPs, DSF@CuO, and DSF@CuO Gel. (**I**,**J**) Rheological analyses were conducted to investigate mechanical properties of DSF@CuO Gel. (**K**) Drug release profile of hydrogel at different pH levels. (**L**,**M**) Hemolysis assessment of DSF@CuO Gel with different concentrations of treatment group. Data are presented as mean ± SD and are representative of three independent experiments.

The DSF@CuO Gel was synthesized following the scheme outlined in Figure 1A. Initially, CuO nanoparticles (NPs) with an average diameter of approximately 150 nm were synthesized using a hydrothermal method [32] (Figure 1B,C). These CuO NPs were dispersed in deionized water, while DSF was dissolved in corn oil, a solvent proved for its high solubility for DSF. Through ultrasonication, a Pickering emulsion named DSF@CuO was successfully formed (Figure S1A), exhibiting a spherical morphology and effectively segregating at the water–oil interface (Figure 1D and Figure S1B,C). Subsequently, a sodium alginate (SA) solution was prepared, into which the DSF@CuO emulsion was incorporated to create a DSF@CuO-SA solution (Figure 1E). Upon the introduction of Ca^2+^ ions, this mixture underwent gelation through coordination with the -COO^−^ groups of SA (Figure 1F).

Scanning electron microscopy (SEM) confirmed the potential efficient drug-loading capacity of the resulting DSF@CuO Gel hydrogel (Figure 1G). Zeta potential measurements for CuO NPs, DSF@CuO, and the DSF@CuO Gel indicated a significant increase upon hydrogel formation, suggesting improved stability and reduced susceptibility to removal (Figure 1H). The mechanical properties of the hydrogels were evaluated through rheological analyses. Frequency sweep tests demonstrated excellent elastic characteristics (G′ consistently greater than G″) for both SA and DSF@CuO Gel hydrogels across various frequencies, indicating structural stability under different conditions (Figure 1I). Notably, DSF@CuO Gel hydrogels exhibited significantly higher G′ and G″ values compared to SA hydrogels, indicative of enhanced mechanical strength due to DSF@CuO inclusion. Strain sweep experiments identified higher strain crossover points for DSF@CuO Gel hydrogels, suggesting superior injectability and drug delivery potential (Figure 1J). This was further validated by successful administration through a 26-gauge needle in our experiments (Figure S1D). Moreover, the DSF@CuO Gel exhibited a gradual release of DSF at pH 6.5, which corresponds to the pH of the HCC TME (Figure 1K). Furthermore, the DSF@CuO Gel demonstrated the ability to transition into a gel state upon subcutaneous injection in mice, under conditions simulating the Ca^2+^ ion concentration in the HCC TME (Figure S2). Hemolysis tests on mouse cardiac blood indicated no coagulation at high concentrations, affirming the biocompatibility of the DSF@CuO Gel (Figure 1L,M). The experimental results above demonstrate that the DSF@CuO Gel is an ideal drug delivery system, capable of sustained drug release in the TME.

### 3.2. Cytotoxicity of DSF@CuO in LenR HCC

**Figure 2:**
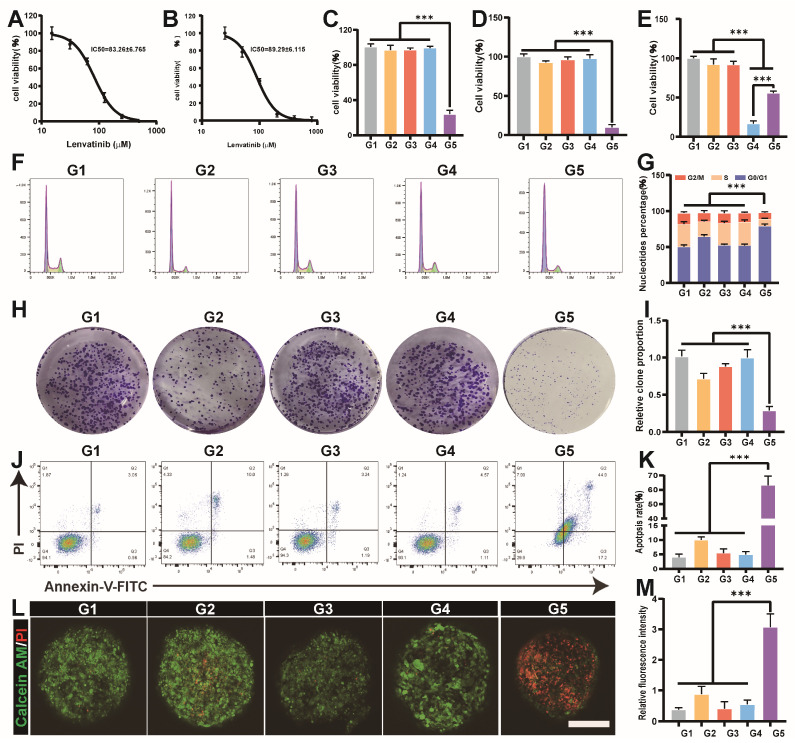
Cytotoxicity of DSF@CuO in LenR HCC. (**A**,**B**) The IC50 for LenR Huh7 and LenR Hep 3B. (**C**–**E**) The CCK-8 assay used to assess the cytotoxic effects of different treatment groups on LenR Huh7, LenR Hep3B, and LO-2. (**F**,**G**) The impact of different treatment groups on cell cycle in LenR Huh7. (**H**,**I**) The impact of different treatment groups on cell clone formation experiment in LenR Huh7. (**J**,**K**) The impact of different treatment groups on apoptosis in LenR-Huh7. (**L**,**M**) The cytotoxic effects of different treatment groups on tumor spheroids in LenR-Huh7 (scale bar = 100 μm). (G1: corn oil, G2: DSF, G3: CuO NPs, G4: Len@CuO, and G5: DSF@CuO). Data are presented as mean ± SD and are representative of three independent experiments. *** *p* < 0.001.

LenR significantly impacts the prognosis of HCC patients. To address LenR, we established stable LenR Huh7 (IC50 = 85.4) and LenR Hep 3B (IC50 = 86.18) cell lines capable of proliferation under high lenvatinib concentrations (Figure 2A,B). Subsequently, the anti-tumor activity of DSF@Cu was explored through CCK-8 assays on LenR HCC cells in vitro. The results showed that DSF@CuO exhibited enhanced cytotoxicity against LenR Huh7, LenR Hep 3B, and Hepa 1-6 cells, with minimal toxicity to the normal liver cell line L-O2, indicating selective action against LenR HCC (Figure 2C–E and Figure S3). The cell cycle can reflect the proliferative potential of cancer cells. Flow cytometry (FCM) results demonstrated that DSF@CuO could induce G0/G1 phase arrest, significantly inhibiting LenR cell proliferation (Figure 2F,G). Colony formation assays further confirmed DSF@CuO’s superior antiproliferative effect, consistent with the CCK8 assay, indicating their antitumor potential (Figure 2H,I). Moreover, we investigated the apoptosis rate of LenR HCC cells using Annexin V-FITC and propidium iodide (PI) double staining methods with different treatment groups. The results indicated that apoptosis rates were notably higher in DSF@CuO-treated LenR Huh7 cells compared to controls (Figure 2J,K). Three-dimensional (3D) tumor sphere models, simulating in vivo TME, provided a more physiologically relevant platform for evaluating DSF@CuO efficacy [33]. The results demonstrate that the largest number of dead cells (red) was observed in the 3D tumor spheroids treated with DSF@CuO, highlighting the potential of DSF@CuO as a therapeutic agent to address the challenges of cancer treatment within the tumor microenvironment (TME) (Figure 2L,M). Taken together, the above study results indicate that DSF@CuO exhibits potent cytotoxic effects against LenR HCC cells in vitro.

### 3.3. DSF@CuO Can Effectively Induce LenR HCC Cuproptosis

**Figure 3:**
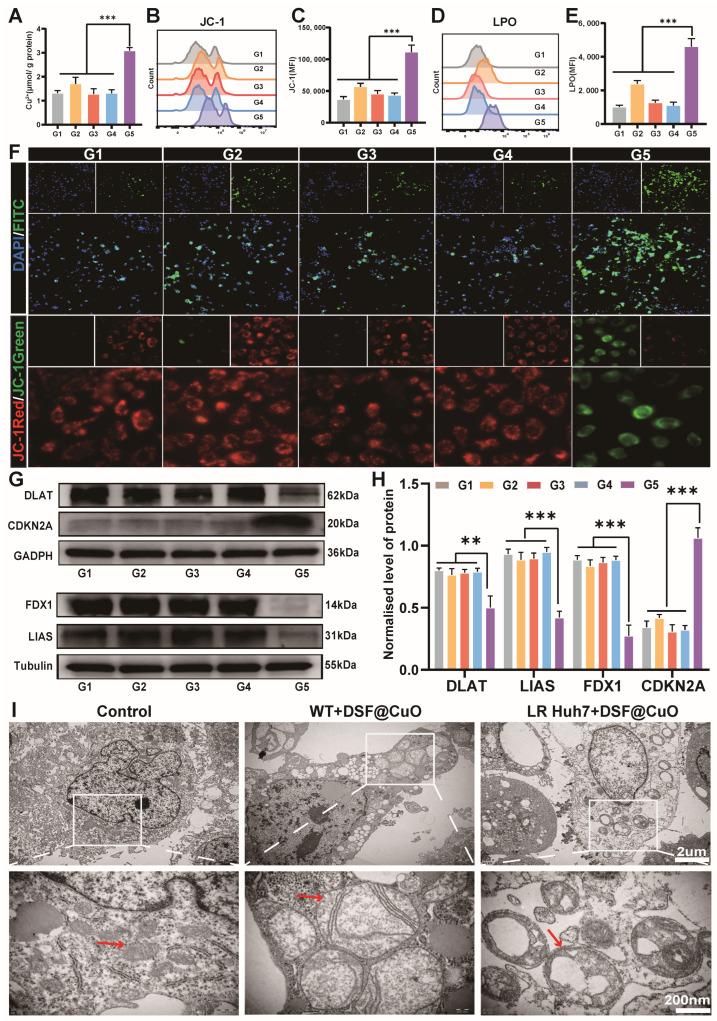
DSF@CuO induced the upregulation of ROS, LPO, and severe mitochondria damage, resulting in cuproptosis. (**A**) The impact of different treatment groups on copper ion transport. (**B**,**C**) FCM was used to analyze the expression of JC-1 in different treatment groups. (**D**,**E**) Flow cytometry was used to analyze the expression of LPO in different treatment groups. (**F**) Fluorescence microscopy is used to detect changes in ROS and JC-1 probe levels in different treatment groups. (**G**,**H**) WB was used to verify the protein expression of cuproptosis across different treatment groups. (**I**) Representative Bio-TEM images of WT-Huh7 cells and LenR HCC cells before and after treatment with DSF@CuO. (G1: corn oil, G2: DSF, G3: CuO NPs, G4: Len@CuO, and G5: DSF@CuO). Data are presented as mean ± SD and are representative of three independent experiments. ** *p* < 0.01, *** *p* < 0.001.

DSF acts as a potent Cu ionophore, and DSF@CuO, with DSF and Cu, was believed to have the strongest ability to transport Cu into tumor cells. To this end, LenR Huh7 cells were treated with corn oil, DSF, CuO NPs, Len@CuO, and DSF@CuO. Our findings demonstrate that DSF@CuO significantly increases intracellular Cu^2+^ concentrations in LenR HCC cells, indicating its potential to induce cuproptosis (Figure 3A). Previous studies have highlighted that cuproptosis primarily involves mitochondrial dysfunction. Mitochondrial dysfunction is typically evaluated by assessing mitochondrial membrane potential, ROS levels, and lipid peroxidation levels [34,35,36]. Flow cytometry and immunofluorescence analyses confirmed that DSF@CuO leads to decreased mitochondrial membrane potential, increased ROS levels, and elevated lipid peroxidation (Figure 3B–F). Western blot (WB) analysis further supported the induction of cuproptosis by DSF@CuO in LenR HCC cells, showing downregulation of anti-cuproptotic proteins (DLAT, LIAS, and FDX1) and upregulation of the pro-cuproptotic protein CDKN2A compared to control groups [37] (Figure 3G,H). TEM provided visual evidence of cuproptosis, revealing characteristic cellular alterations such as nuclear pyknosis, vacuolization of the endoplasmic reticulum, severe mitochondrial swelling, fragmentation of mitochondrial cristae, and rupture of mitochondrial membranes in some cells (Figure 3I). Furthermore, analysis of clinical data from HCC patients in the TCGA database reveals a robust correlation between the cuproptosis gene set and the HCC cancer stemness gene set, thereby further validating the potential of cuproptosis as a therapeutic approach for HCC patients (Figure S4). The inhibition of cell stemness characteristics will be validated in the subsequent section. All in all, this emerging cell death pathway, cuproptosis, exhibits distinctive ultrastructural features that distinguish it from ferroptosis and apoptosis, suggesting its potential as a promising therapeutic approach for LenR HCC treatment.

### 3.4. DSF@CuO Could Inhibit the Cell Stemness of LenR HCC

**Figure 4:**
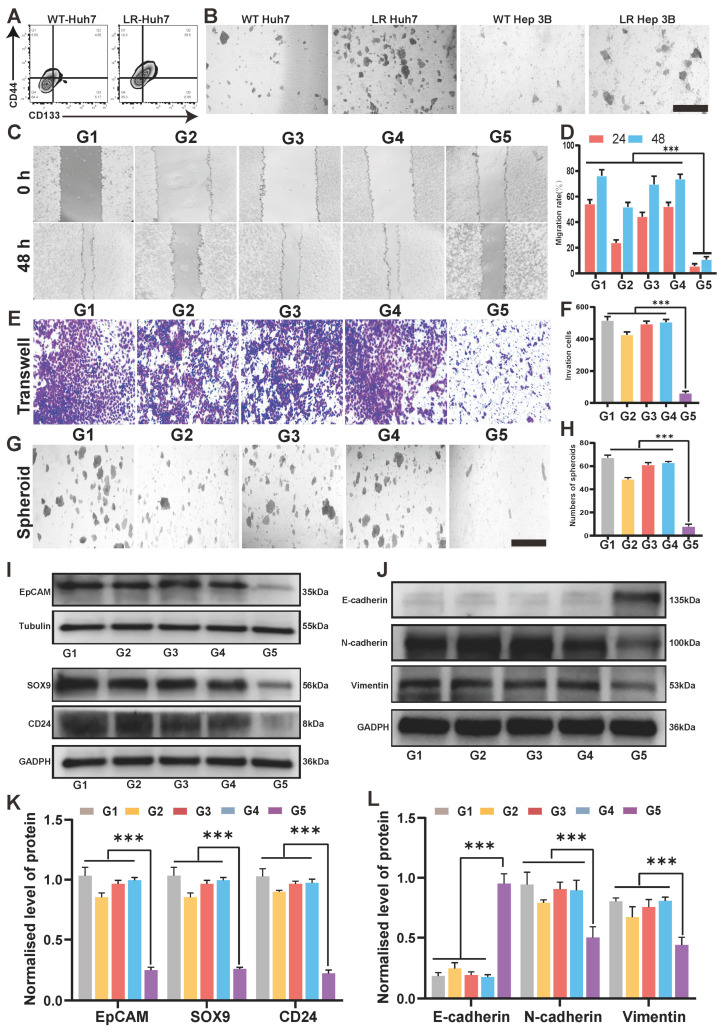
DSF@CuO could suppress cell stemness-related characteristics in LenR HCC. (**A**) The expression of CD44 and CD133 in WT-Huh7 and LenR-Huh7. (**B**) Tumor sphere-forming capacity in both LenR cell lines and the WT (Scale bar = 50 μm). (**C**,**D**) A wound-healing assay showing the migration of corn oil, DSF, CuO NPs, lenvatinib, and DSF@CuO-treated LenR Huh7 cells in 0, 24, and 48 h. (**E**,**F**) A transwell assay was used to evaluate the impact of different treatment groups on the invasion ability of LenR Huh7. (**G**,**H**) The impact of different treatment groups on tumor spheroid formation in LenR-Huh7 (scale bar = 50 μm). (**I**,**K**) Western blot analysis of EpCAM, SOX9, and CD24 protein levels in LenR Huh7 cells after incubation with different groups. (**J**,**L**) Western blot analysis of E-cadherin, N-cadherin, and Vimentin protein levels in LenR Huh7 cells after incubation with different groups. (G1: corn oil, G2: DSF, G3: CuO NPs, G4: Len@CuO, and G5: DSF@CuO). Data are presented as mean ± SD and are representative of three independent experiments. *** *p* < 0.001.

Recently studies have highlighted stemness as a primary contributor to LenR in HCC [8,38,39]. The ability of tumor cells to form spheroids reflects their potential cancer stemness [40]. Flow cytometry (FCM) analysis revealed elevated expressions of CD133 and CD44, markers representing HCC cancer stemness [41,42], in both LenR cell lines compared to their wild-type (WT) counterparts (Figure 4A and Figure S5), underscoring the significant role of cancer stemness in LenR HCC. Additionally, the LenR cell lines exhibited significantly enhanced spheroid-forming ability compared to WT cell lines (Figure 4B). Therefore, this study will verify the inhibitory effect of DSF@CuO on cell stemness in LenR HCC through subsequent experiments. Epithelial–mesenchymal transition (EMT) represents the ability of tumor cells to invade and metastasize, and it is also a critical manifestation of cancer stemness [43]. Migration and wound-healing assays are commonly used indicators to assess the EMT status of tumor cells. Research results have shown that DSF@CuO significantly inhibited the migratory and invasive abilities of LenR Huh7 cells (Figure 4C–F). Furthermore, DSF@CuO treatment significantly reduced both the rate of sphere formation and the size of spheres in the LenR HCC cell lines, demonstrating its potent inhibitory effect on tumor stemness (Figure 4G and Figure S6). The protein expression levels of SOX9 and CD24 are important indicators that reflect the strength of stemness in HCC cells [44,45]. Western blot (WB) results further showed that DSF@CuO significantly downregulated the expression of molecules associated with HCC cancer stemness (Figure 4I) and inhibited EMT, thereby suppressing the cell stemness of LenR HCC (Figure 4J).

### 3.5. The Mechanism of DSF@CuO in Overcoming LenR HCC

**Figure 5:**
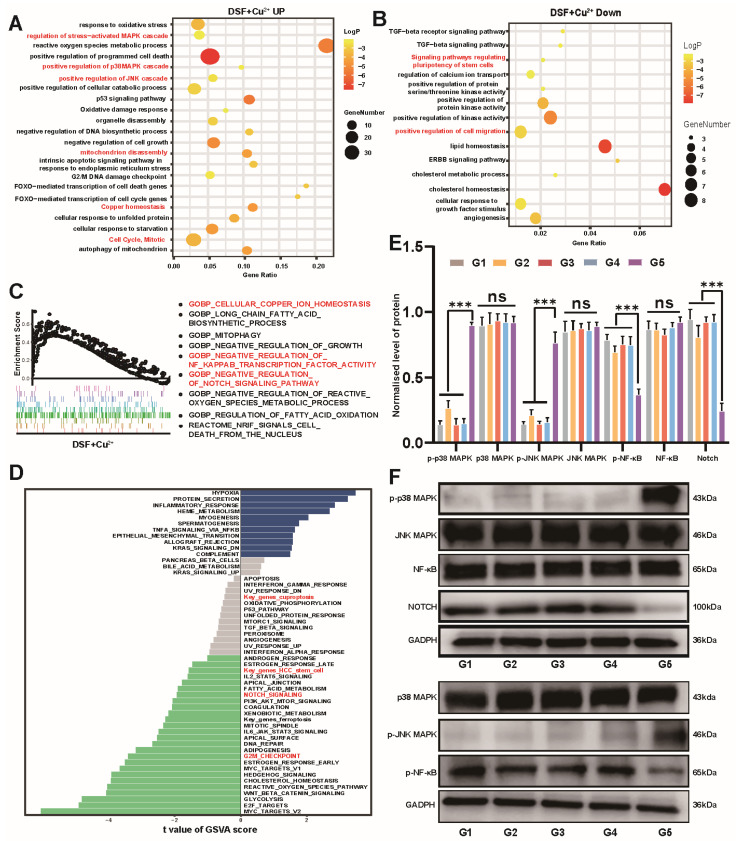
Mechanisms of DSF@CuO in inhibiting cell stemness of LenR HCC. (**A**,**B**) GO and KEGG enrichment analysis of DEGs in DSF+Cu^2+^ group. (**C**) GSEA plots showing pathways upregulated and downregulated after DSF+Cu^2+^ treatment. (**D**) GSVA plots showing pathways upregulated and downregulated after DSF treatment. (**E**,**F**) Western blot analysis of p-p38MAPK, p38MAPK, p-JNK MAPK, JNK MAPK, p-NF-Κb, NF-Κb, and NOTCH protein levels in LenR Huh7 cells after incubation with different groups. (G1: corn oil, G2: DSF, G3: CuO NPs, G4: Len@CuO, and G5: DSF@CuO). Data are presented as mean ± SD and are representative of three independent experiments. *** *p* < 0.001, ns, not significant.

To further investigate the mechanisms of action of DSF and Cu on cancer cells, transcriptional data of PBS, DSF, and DSF combined with Cu^2+^ treated on HCC Huh7 cell lines were downloaded from the GEO database. Differential gene clustering results highlighted that genes affected in the combination group primarily involve cuproptosis, cancer stemness, and invasion and metastasis capabilities (Figures S7–S9). Principal Component Analysis (PCA) was employed to elucidate the variance between the groups, highlighting a more distinct separation in the combined treatment group compared to the DSF monotherapy group, indicating a synergistic interaction between DSF and Cu^2+^ at the transcriptomic level and the reliability of the RNA-Seq data (Figures S7 and S8). Next, integrating insights from three enrichment analyses, we found that the synergistic effect of DSF combined with Cu^2+^ predominantly operates by upregulating the JNK/p38 MAPK signaling pathways [46,47] and downregulating the NF-κB and NOTCH pathways [48], crucial pathways in cancer stemness (Figure 5A,D and Figures S9–S11). This comprehensive analysis underscores the therapeutic potential of DSF@CuO for treating LenR HCC, providing novel strategies to combat cancer stemness. Next, we validated these findings using WB to confirm the pathways identified through bioinformatics screening. The results demonstrated that DSF@CuO primarily targets LenR HCC by activating the JNK/p38 MAPK pathway and inhibiting the NF-κB and NOTCH signaling pathway (Figure 5E,F). This integrated approach suggests a promising strategy for overcoming LenR in HCC by targeting cancer stemness-related pathways.

### 3.6. DSF@CuO Gel Could Overcome LenR In Vivo

**Figure 6:**
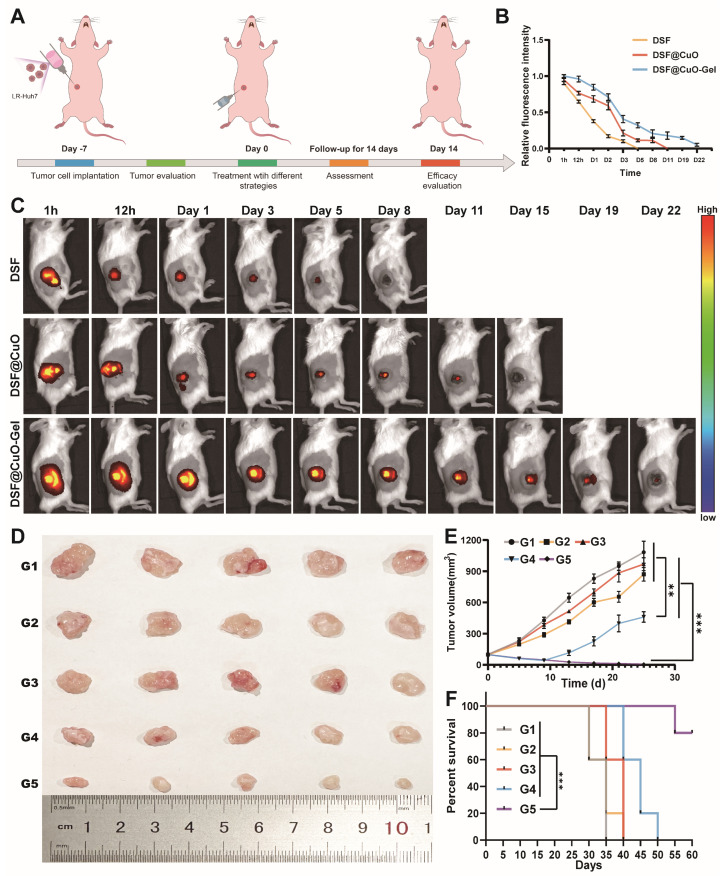
DSF@CuO Gel could inhibit tumor growth in LenR mice models. (**A**) Schematic illustrating treatment procedure. (**B**) Quantitative evaluation of sustained release effects of DSF, DSF@CuO, and DSF@CuO Gel in mouse models (n = 3). (**C**) IVIS results for evaluating retention behavior of DSF, DSF@CuO, and DSF@CuO Gel hydrogel after intratumor injection. (**D**) Photographs of dissected mouse tumor tissues after completion of treatment (n = 5). (**E**) In vivo tumor volume curve of each group of mice during treatment process. (**F**) Survival analysis of mice across different treatment groups. (G1: sodium alginate, G2: DSF, G3: CuO NPs, G4: DSF@CuO, and G5: DSF@CuO Gel). Data are presented as mean ± SD and are representative of three independent experiments. ** *p* < 0.01, *** *p* < 0.001.

Based on the promising therapeutic efficacy observed in in vitro cellular experiments, this study progressed to validate these treatment effects in vivo. Initially, we established a subcutaneous model of LenR HCC in nude mice and conducted treatments along with relevant assessments (Figure 6A). We then investigated the sustained-release capabilities of the DSF@CuO Gel hydrogel in mouse tumors, revealing its complete degradation within approximately 22 days, contrasting with DSF@CuO emulsion (11 days) and separately injected DSF (5 days), highlighting the hydrogel’s enhanced drug retention and sustained release properties (Figure 6B,C). For treatment evaluation, twenty-five mice were randomly assigned to five groups: SA, DSF, CuO NPs, DSF@CuO, and DSF@CuO Gel. The DSF@CuO treatment initially led to a decrease in tumor volume followed by an increase, whereas the DSF@CuO Gel group showed sustained tumor volume reduction, indicating superior therapeutic outcomes due to the hydrogel’s sustained release effect (Figure 6D,E). Survival analysis further demonstrated a significant improvement in the survival rate of the DSF@CuO Gel-treated group, with 80% of the mice surviving up to 60 days post-treatment, contrasting with outcomes in other groups (Figure 6F). This highlights its potential as a significant advancement in LenR HCC treatment and offers a promising clinical strategy for patients with advanced HCC.

### 3.7. DSF@CuO Gel Could Overcome In Situ HCC Model

**Figure 7:**
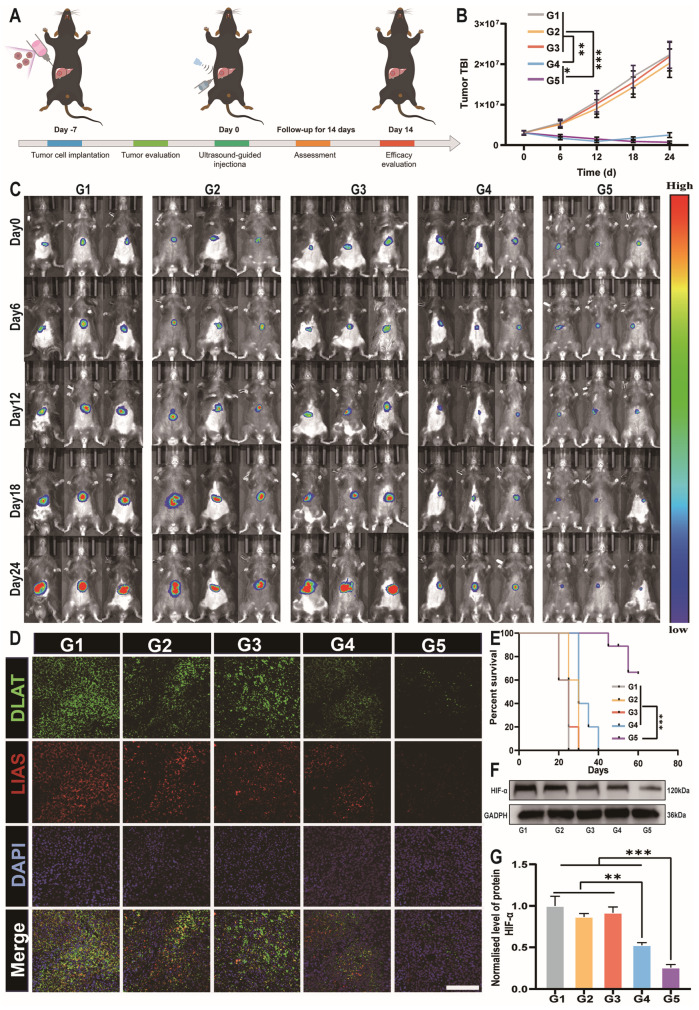
DSF@CuO Gels could inhibit tumors in an in situ mice model of HCC. (**A**) A schematic illustrating the treatment procedure. (**B**,**C**) IVIS for monitoring the size of in situ HCC in mice. (**D**) Immunofluorescence images of cuproptosis proteins in mouse tumor tissues after treatment across different groups; cell nuclei were stained with DAPI (blue), the DLAT were stained with FITC (green), and the LIAS were stained with cy3 (green) (scale bars = 20 μm). (**E**) Survival analysis of mice during the treatment period across different treatment groups. (**F**,**G**) WB detection of HIF-1α expression. (G1: sodium alginate, G2: DSF, G3: CuO NPs, G4: DSF@CuO, and G5: DSF@CuO Gel). Data are presented as mean ± SD and are representative of three independent experiments. * *p* < 0.05, ** *p* < 0.001, *** *p* < 0.001.

To validate the therapeutic efficacy of the DSF@CuO Gel in a more complex liver TME, this study established a liver orthotopic tumor model in vivo (Figure 7A). Ultrasound guidance was employed to precisely localize tumors within the liver, enhancing the accuracy of our experimental setup (Figure S12). Next, we used the IVIS system to monitor tumor volumes in mice from different treatment groups. While DSF@CuO initially reduced tumor volume, subsequent regrowth underscored the limitations of non-sustained-release formulations. The continuous decrease in tumor volume observed in the DSF@CuO Gel treatment group of mice may be attributed to the sustained release of the hydrogel (Figure 7B,C). Body weight measurements taken every three days following in situ injection of the DSF@CuO Gel into the liver showed no significant differences across treatment groups (Figure S13). Immunofluorescence analyses of post-treatment tumor tissues revealed a marked decrease in the expression of key cuproptosis-related proteins, DLAT and LIAS, in the DSF@CuO Gel group compared to controls (Figure 7D and Figure S15A,C). Survival analysis further supported the effectiveness of the DSF@CuO Gel, with a significantly higher survival rate observed compared to other treatments (Figure 7E). HIF-α is a crucial gene regulating the glycolytic pathway, promoting glucose uptake and lactate accumulation. Furthermore, DSF@CuO Gel treatment alleviated the acidic TME, leading to reduced levels of HIF-α in the TME, indicating potential inhibition of the tumor resistance pathway (Figure 7F,G) [49]. In conclusion, the DSF@CuO Gel effectively induces cuproptosis in an orthotopic HCC model while suppressing the HIF-α-mediated drug resistance pathway. This dual action significantly inhibits tumor growth and improves survival rates in mice, highlighting its potential as an advanced therapeutic strategy for treating advanced HCC.

### 3.8. Biosafety Evaluation of DSF@CuO Gel

Finally, we evaluated the biological safety of the DSF@CuO Gel through a comprehensive biotoxicity analysis to assess its potential for clinical translation. Human umbilical vein endothelial cells (HUVECs) are currently the most commonly used cells for evaluating the cytotoxicity of nanomaterials [50]. The CCK8 assay assessed the cell viability of HUVECs after treatment, demonstrating favorable biosafety of the DSF@CuO Gel (Figure S16A). Evaluation of potential foreign body response included measuring IL-4 expression levels in tumor tissues [51]. The results indicated no significant inflammatory response compared to the control, confirming the biocompatibility of the hydrogel (Figure S16B). Additionally, we monitored the weight of mice post in situ injection treatment and conducted histological examinations of major organs, liver function, and kidney function at the end of the treatment period. As shown in Figure S17A–F, the DSF@CuO Gel exhibited no significant biotoxicity compared to the control group. These findings highlight the excellent biological safety and biocompatibility of the DSF@CuO Gel, underscoring its potential for further clinical translation research.

## 4. Conclusions

We developed a DSF@CuO Gel, an injectable hydrogel designed to reverse LenR HCC. This hydrogel offers excellent injectability, biocompatibility, and rapid in situ gelation within tumors. It provides sustained release of Cu^2+^ in response to the acidic TME, ensuring high concentrations of DSF and Cu^2+^ in tumor tissues while minimizing systemic toxicity. In vitro, the DSF@CuO Gel targets LenR HCC cells by activating the p38/JNK MAPK pathway and inhibiting NF-κB and NOTCH pathways, causing significant mitochondrial and cellular damage. In vivo, it effectively inhibits tumor growth in both subcutaneous and in situ LenR models. The DSF@CuO Gel shows promising antitumor effects, presenting a novel therapeutic approach for treating LenR HCC.

## Data Availability

The original contributions presented in the study are included in the article/Supplementary Material, further inquiries can be directed to the corresponding author.

## Supplementary Materials

The following supporting information can be downloaded at: https://www.mdpi.com/article/10.3390/polym16172418/s1, Figure S1: Characterization of injectable hydrogel DSF@CuO Gel. Figure S2: DSF@CuO-Gel Gelation behavior in vivo after subcutaneous injection in mice. Figure S3. CCK-8 assay used to assess the cytotoxic effects of different treatment groups on LR Hep 3B. Figure S4. The correlation analysis between key cuproptosis genes and genes associated with effector T cells. Figure S5. The proportion of CSCs in Hep 3B WT and LR strains. Figure S6. The impact of different treatment groups on the sphere formation ability of LR Hep 3B tumors. Figure S7. Volcano and PCA plot of gene expression of upregulated and downregulated genes between PBS and DSF groups. Figure S8. Volcano and PCA plot of gene expression of upregulated and downregulated genes between PBS and DSF+Cu^2+^ groups. Figure S9. A heatmap analysis DEGs among different treatment groups. Figure S10. GO and KEGG enrichment analysis of DEGs in DSF group. Figure S11. GSEA plots showing pathways upregulated and downregulated after DSF treatment. Figure S12. GSVA plots showing pathways upregulated and downregulated after DSF treatment. Figure S13. Under ultrasound guidance, the injectable hydrogel DSF@CuO-Gel is used for the treatment of liver orthotopic tumors. Figure S14. Graph of mice weight changes in different treatment groups. Figure S15. Statistical chart of DLAT and LIAS levels in tumor tissues after treatment in different groups. Figure S16. Foreign body assessment in different treatment groups. Figure S17. Biological safety assessment of DSF@CuO Gel.
