# Supplementary material for: Injectable Hydrogel-Encapsulating Pickering Emulsion for Overcoming Lenvatinib-Resistant Hepatocellular Carcinoma via Cuproptosis Induction and Stemness Inhibition
Source: Polymers (Basel). 2024 Aug 26;16(17):2418. doi: 10.3390/polym16172418 (PMC11397159; doi:10.3390/polym16172418)
Supplement: Supplementary file 1 [file polymers-16-02418-s001.zip › polymers-3146781-supplementary.pdf]

Supplementary Information for:

# Injectable Hydrogel-Encapsulating Pickering Emulsion for Overcoming Lenvatinib-Resistant Hepatocellular Carcinoma via Cuproptosis Induction and Stemness Inhibition

**Xin Li [1,†], Chuanyu Tang [1,†], Hanjie Ye [1] and Chihua Fang [1,2,3,4,*]**

[1] Department of Hepatobiliary Surgery I, General Surgery Center, Zhujiang Hospital,
Southern Medical University, Guangzhou 510280, China;
lixinlucky1314@163.com (X.L.); chuanyutang1932@163.com (C.T.);
yehanjie1978@163.com (H.Y.)

[2] Institute of Digital Intelligent Minimally Invasive Surger, Zhujiang Hospital, Southern Medical University, Guangzhou 510280, China

[3] Guangdong Provincial Clinical and Engineering Center of Digital Medicine, Guangzhou 510280, China

[4] South China Institute of National Engineering Research Center of Innovation and Application of Minimally Invasive Instruments, Guangzhou 510280, China

* Correspondence: fangchihua@smu.edu.cn

† These authors contributed equally to this work.

## Supplementary Figure

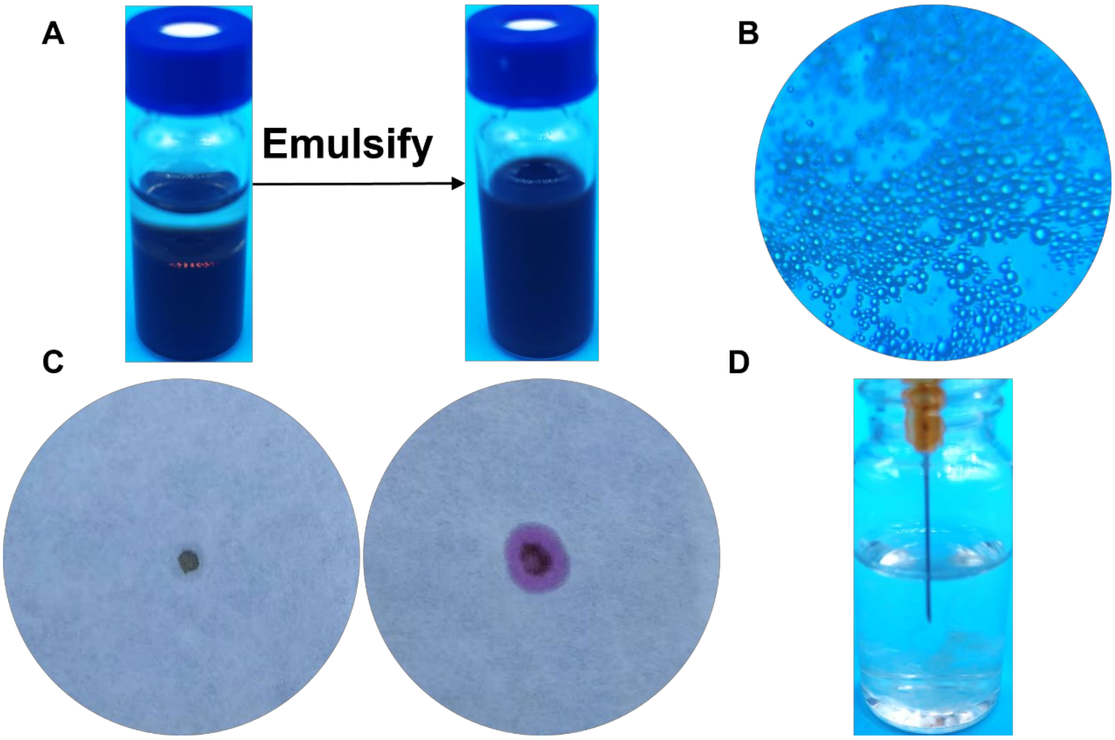


**Figure S1.** Characterization of injectable hydrogel DSF@CuO Gel. A) Photos before and after the formation of DSF@CuO; B) Images of DSF@CuO under a conventional optical microscope; C) Disk diffusion method used to characterize the morphology of DSF@CuO; D) Needle penetration test of DSF@CuO Gel in calcium ion solution

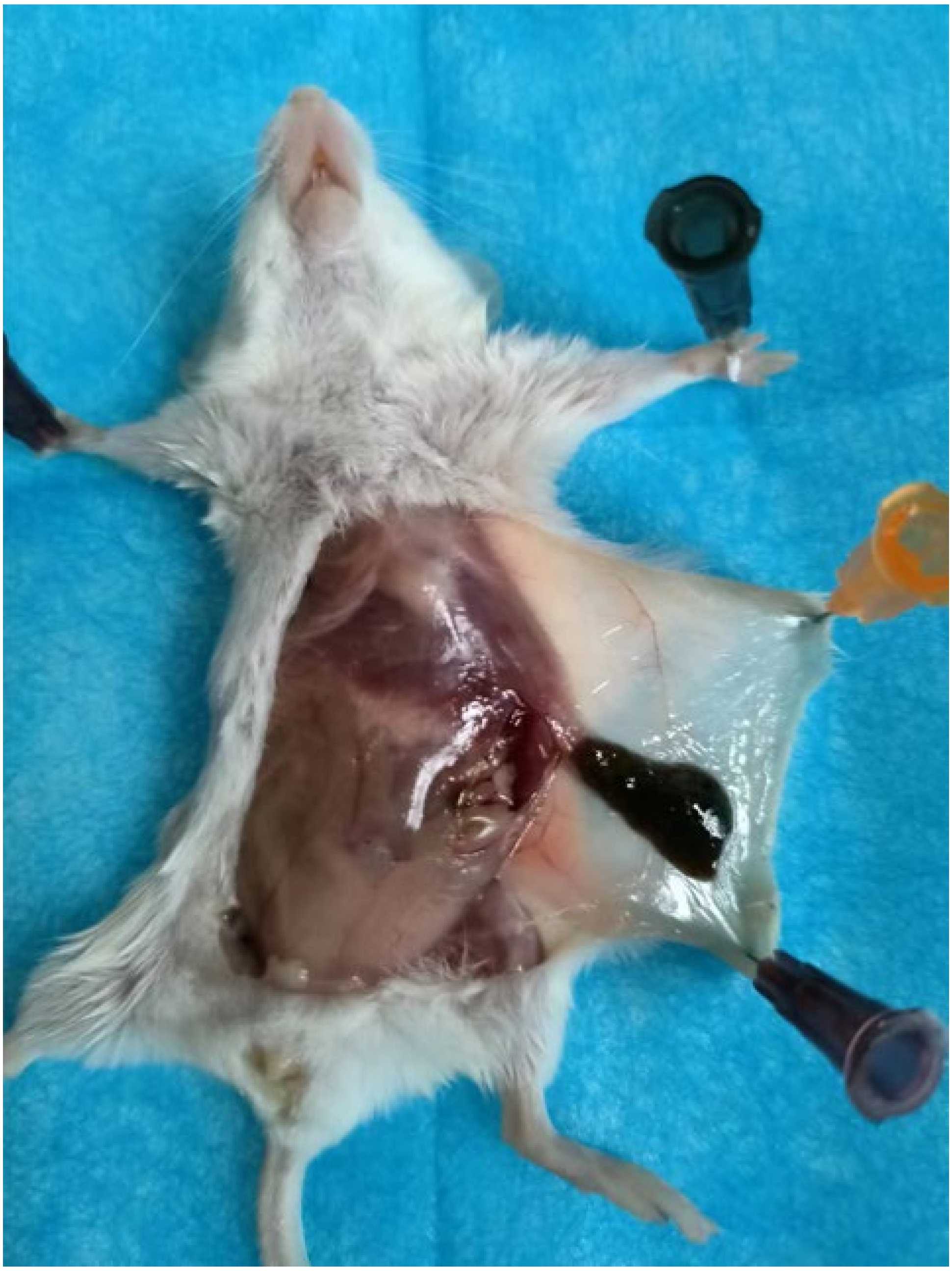

**Figure S2.** DSF@CuO Gel Gelation behavior in vivo after subcutaneous injection in mice.

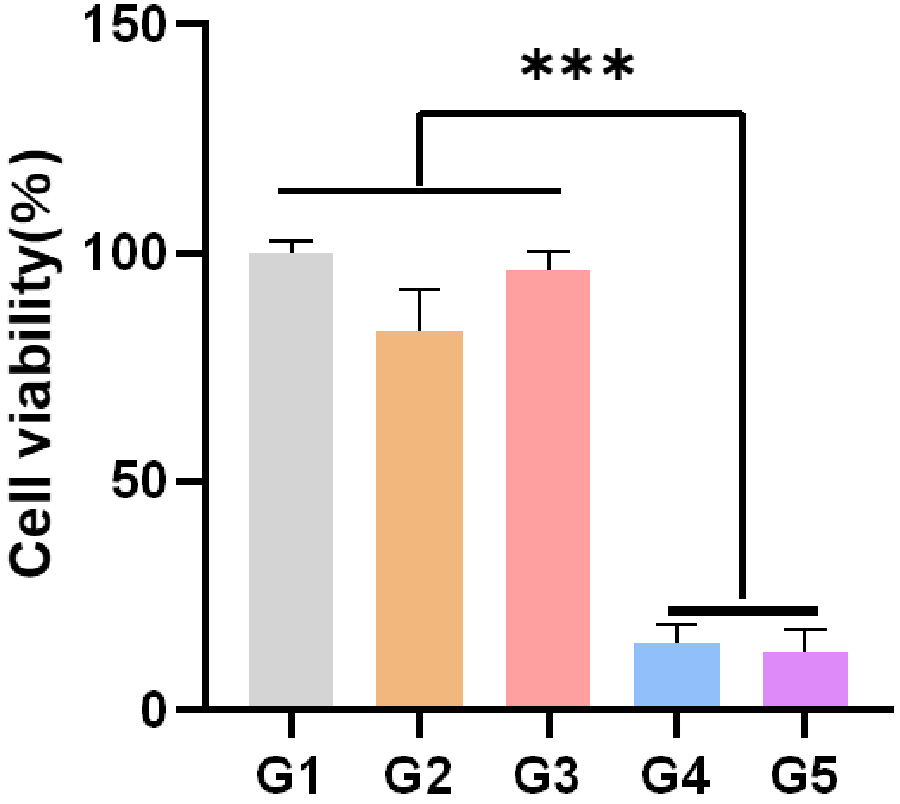


**Figure S3.** CCK-8 assay used to assess the cytotoxic effects of different treatment groups on LR Hep 3B. (G1: corn oil, G2: DSF, G3: CuO NPs, G4: Len@CuO, and G5: DSF@CuO). Data are presented as mean ± SD and are representative of three independent experiments. ***$p < 0.001$.

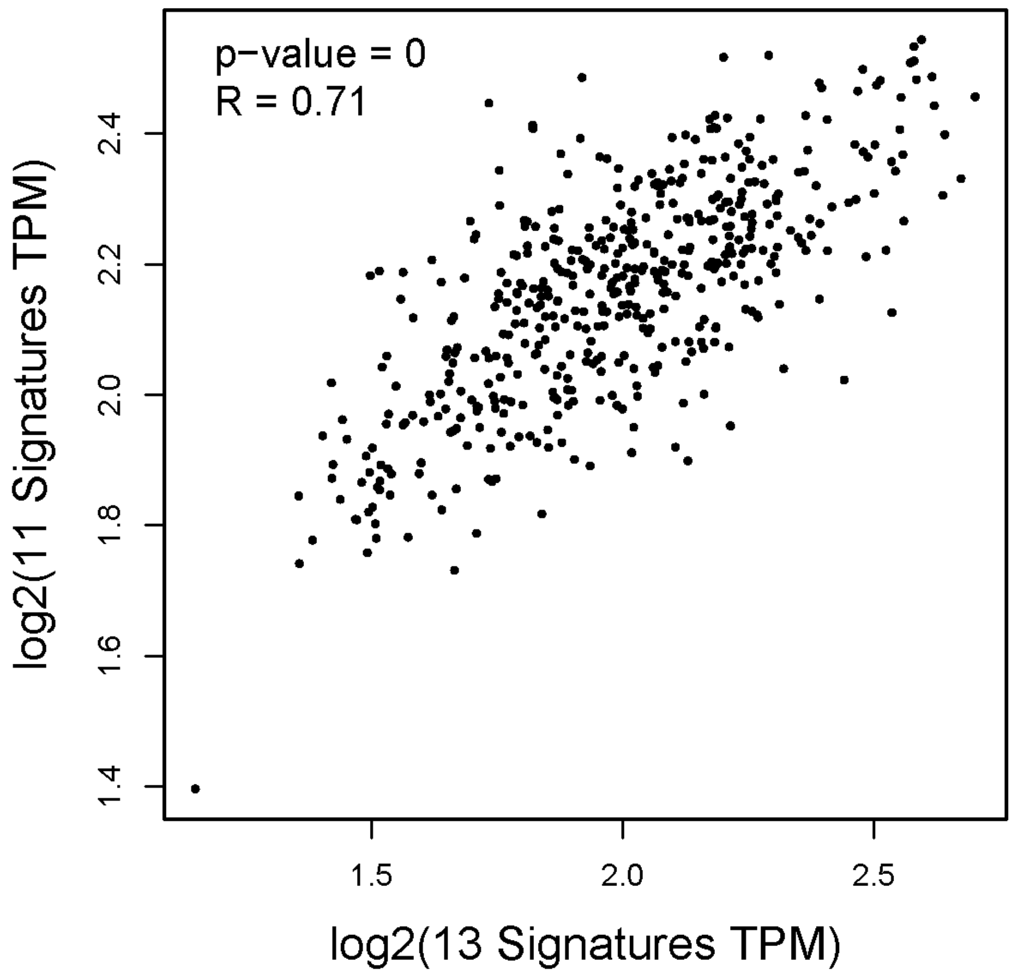


**Figure S4.** The correlation analysis between key cuproptosis genes and genes associated with effector T cells.

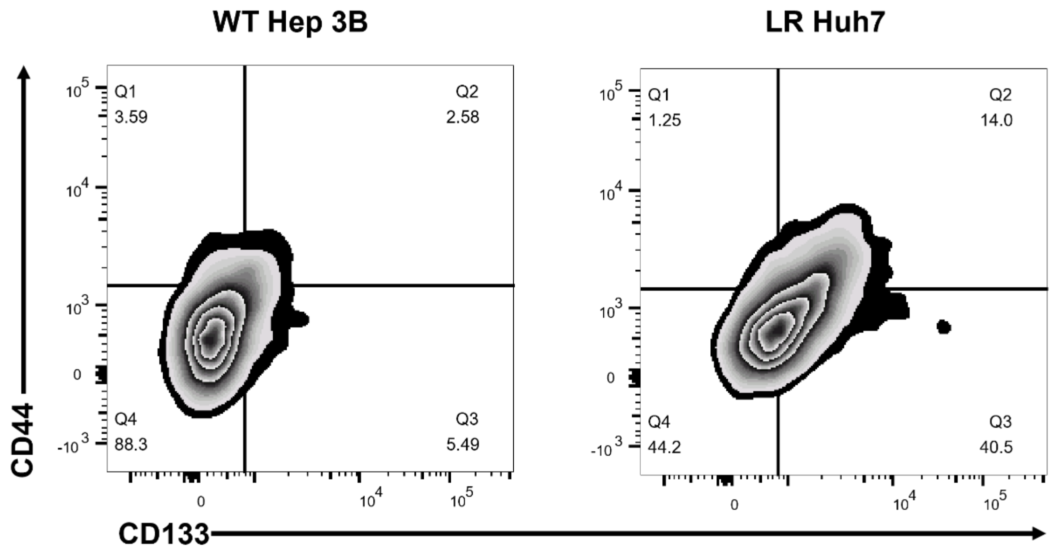


**Figure S5.** The proportion of CSCs in Hep 3B WT and LR strains.

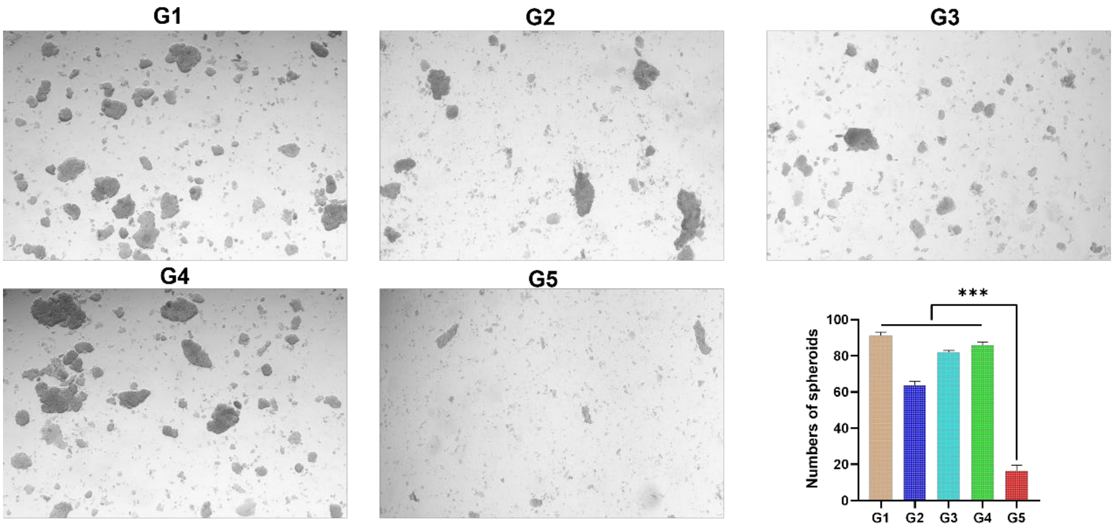


**Figure S6.** The impact of different treatment groups on the sphere formation ability of LR Hep 3B tumors. (G1: corn oil, G2: DSF, G3: CuO NPs, G4: Len@CuO, and G5: DSF@CuO). Data are presented as mean ± SD and are representative of three independent experiments. ***$p < 0.001$.

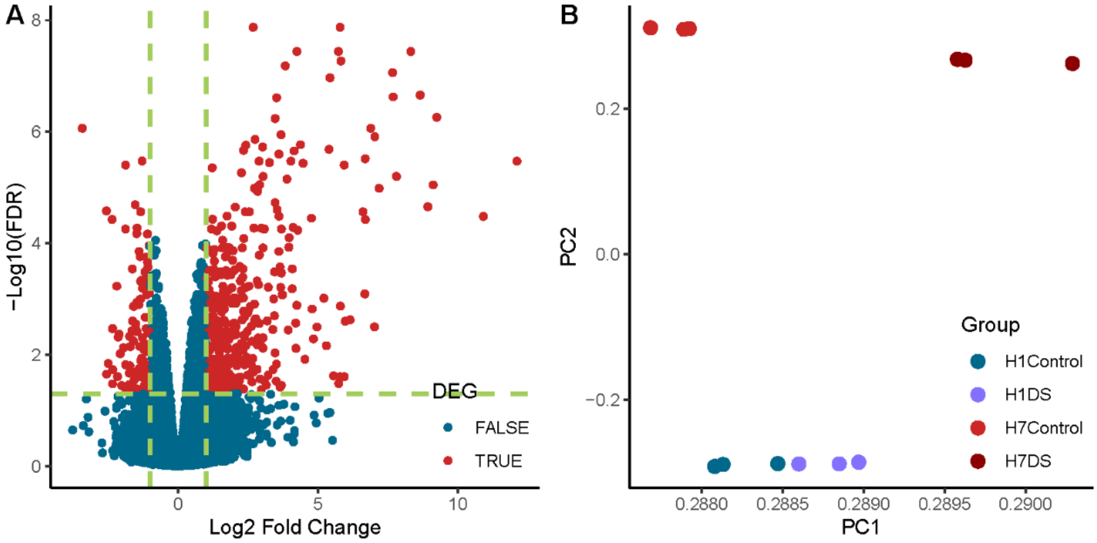


**Figure S7.** Volcano and PCA plot of gene expression of upregulated and downregulated genes between PBS and DSF groups (log2FC≥2, FDR DSF≤ 0.5).

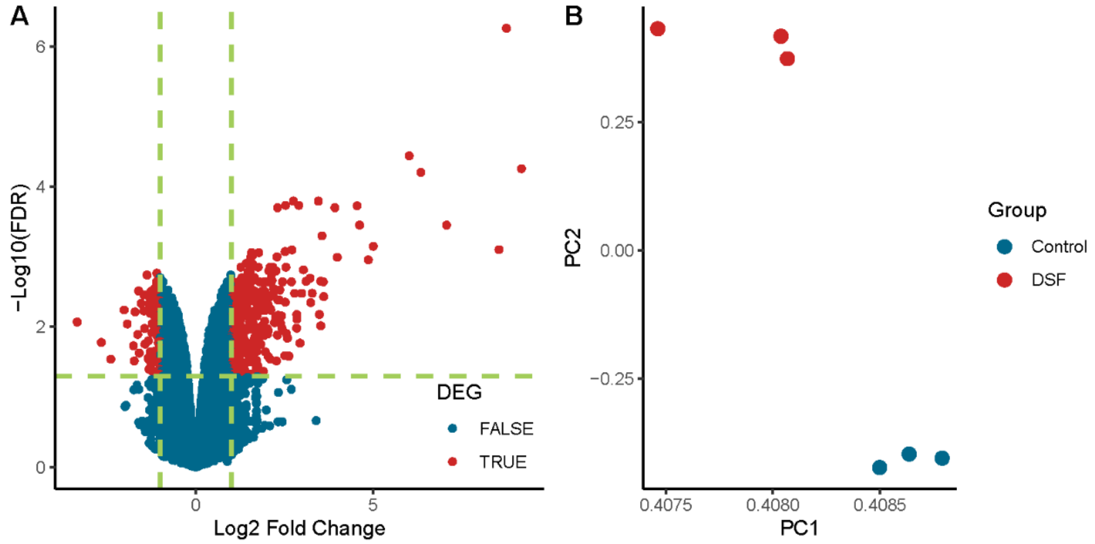


**Figure S8.** Volcano and PCA plot of gene expression of upregulated and downregulated genes between PBS and DSF+$Cu^{2+}$ groups (log2FC≥2, FDR ≤ 0.5)

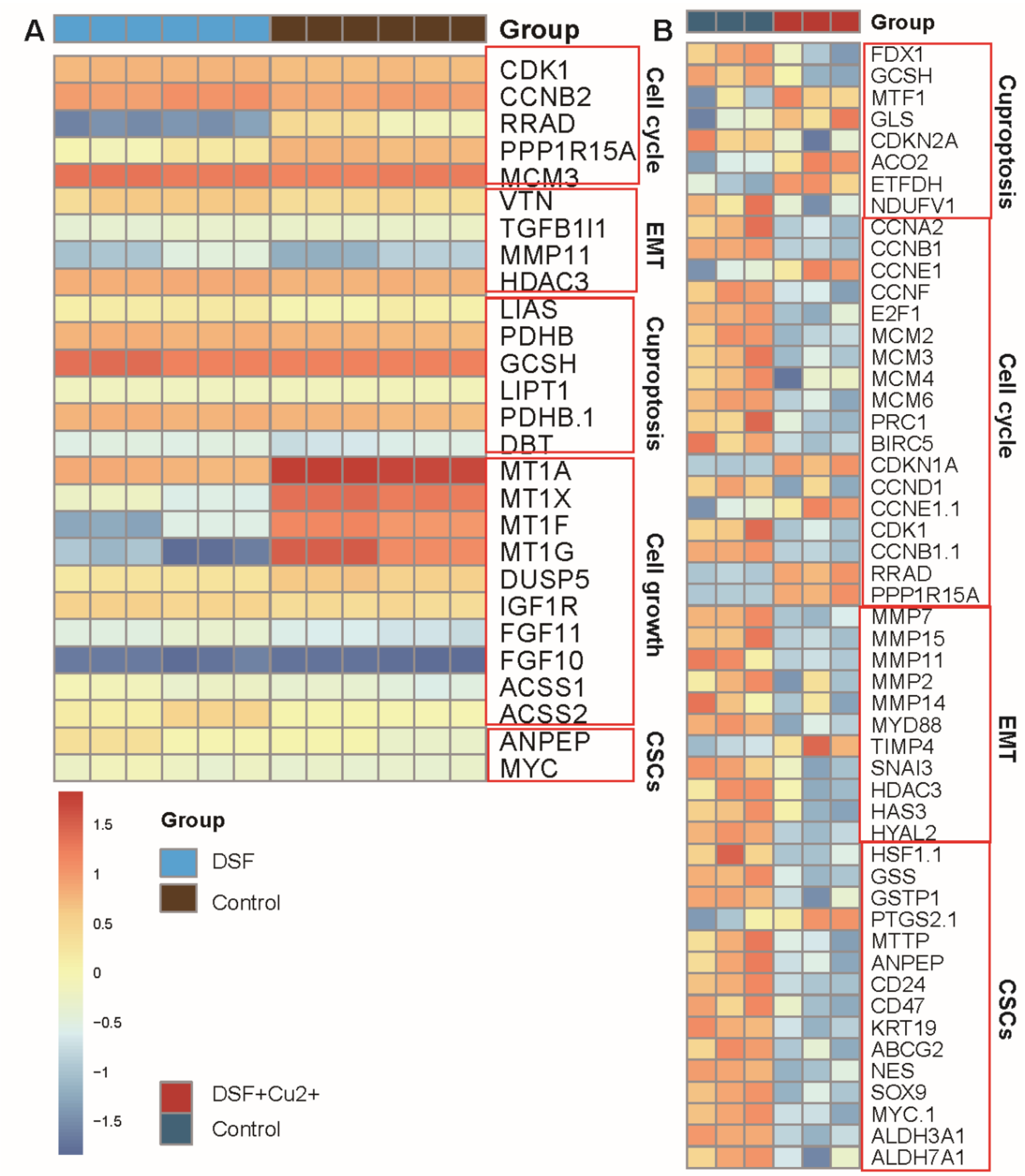


**Figure S9.** A heatmap analysis DEGs among different treatment groups.

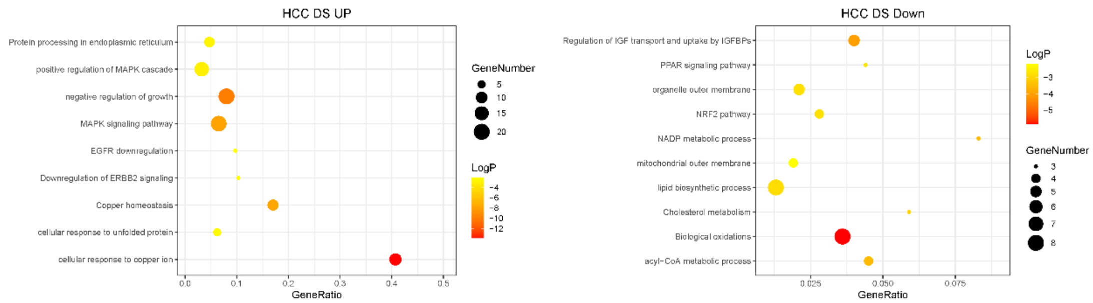


**Figure S10.** GO and KEGG enrichment analysis of DEGs in DSF group.

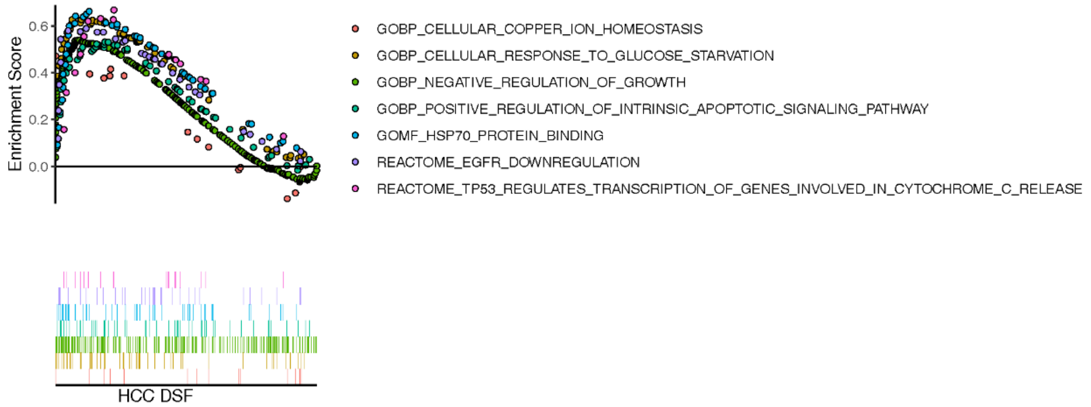


**Figure S11.** GSEA plots showing pathways upregulated and downregulated after DSF treatment.

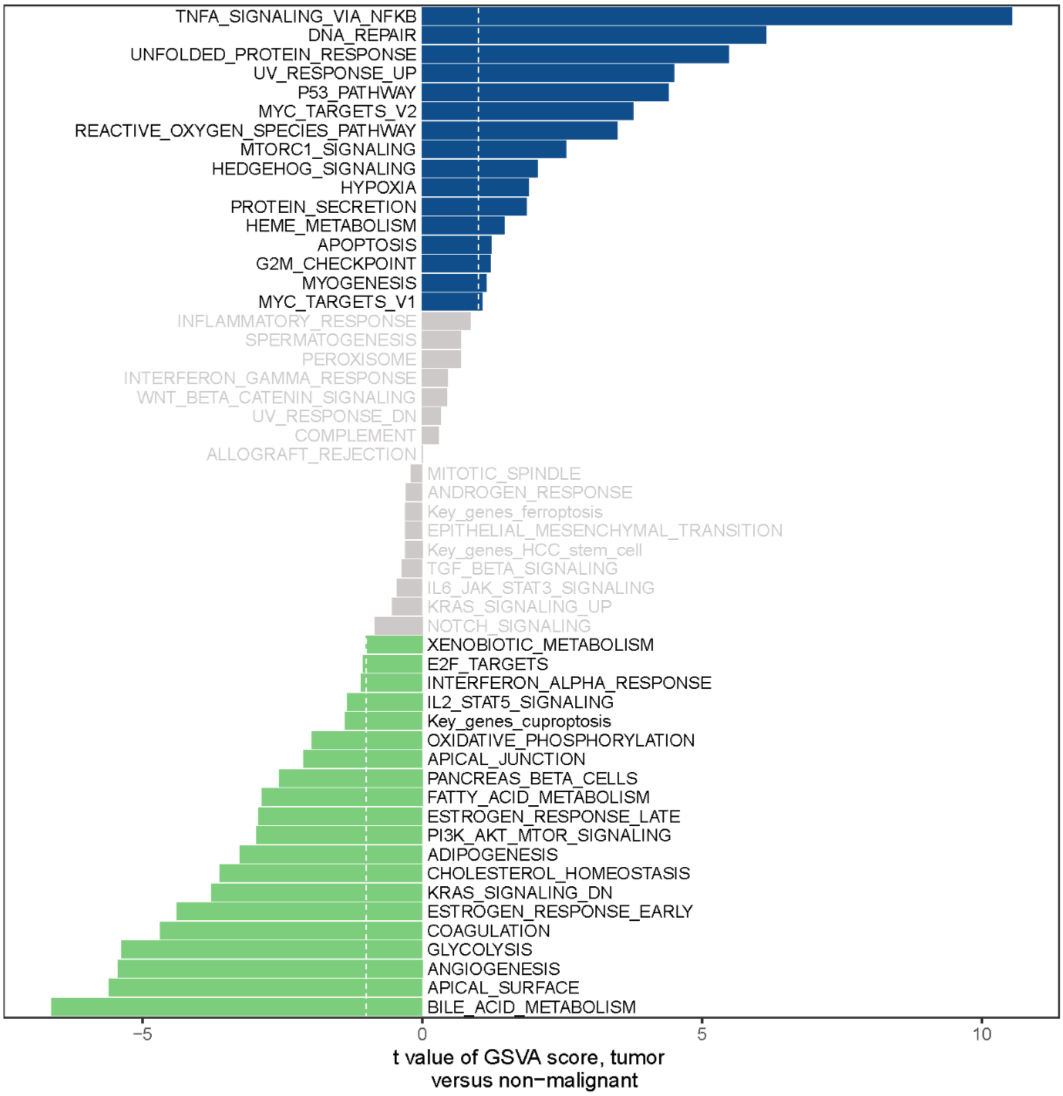


**Figure S12.** GSVA plots showing pathways upregulated and downregulated after DSF treatment

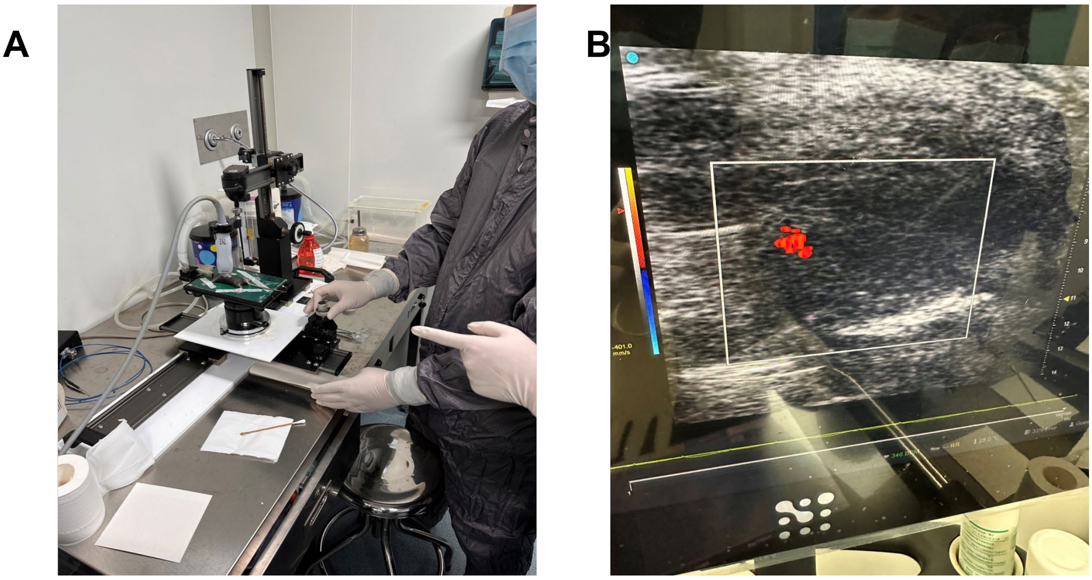


**Figure S13.** Under ultrasound guidance, the injectable hydrogel DSF@CuO-Gel is used for the treatment of liver orthotopic tumors.

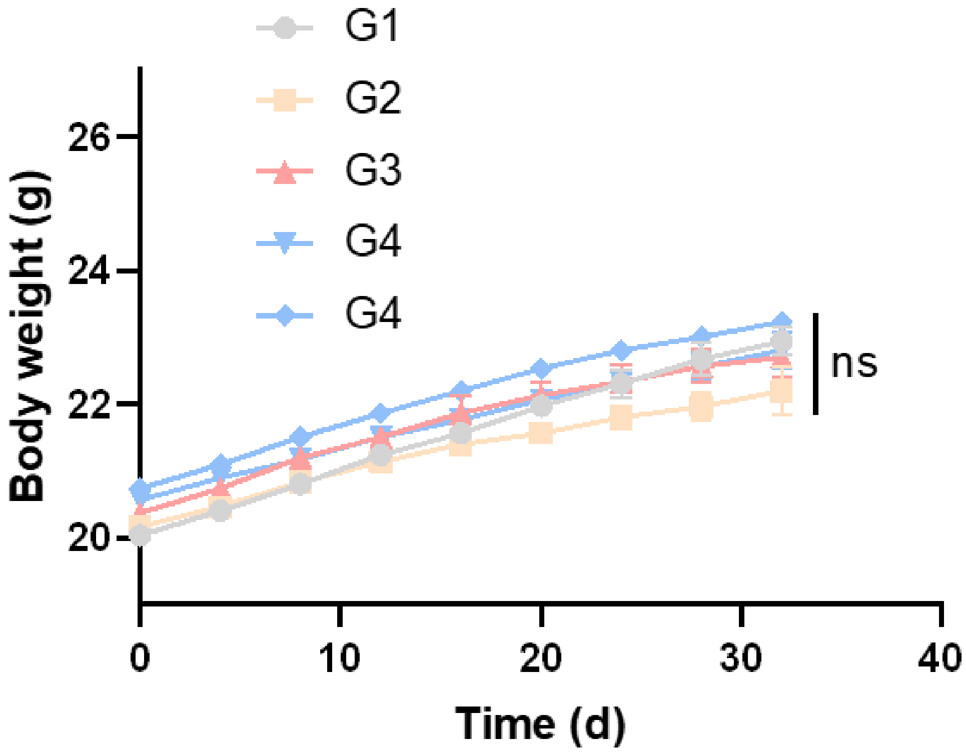


**Figure S14.** Graph of mice weight changes in different treatment groups. (G1: sodium alginate, G2: DSF, G3: CuO NPs, G4: DSF@CuO, and G5: DSF@CuO Gel). Data are presented as mean ± SD and are representative of three independent experiments. ns: no significance.

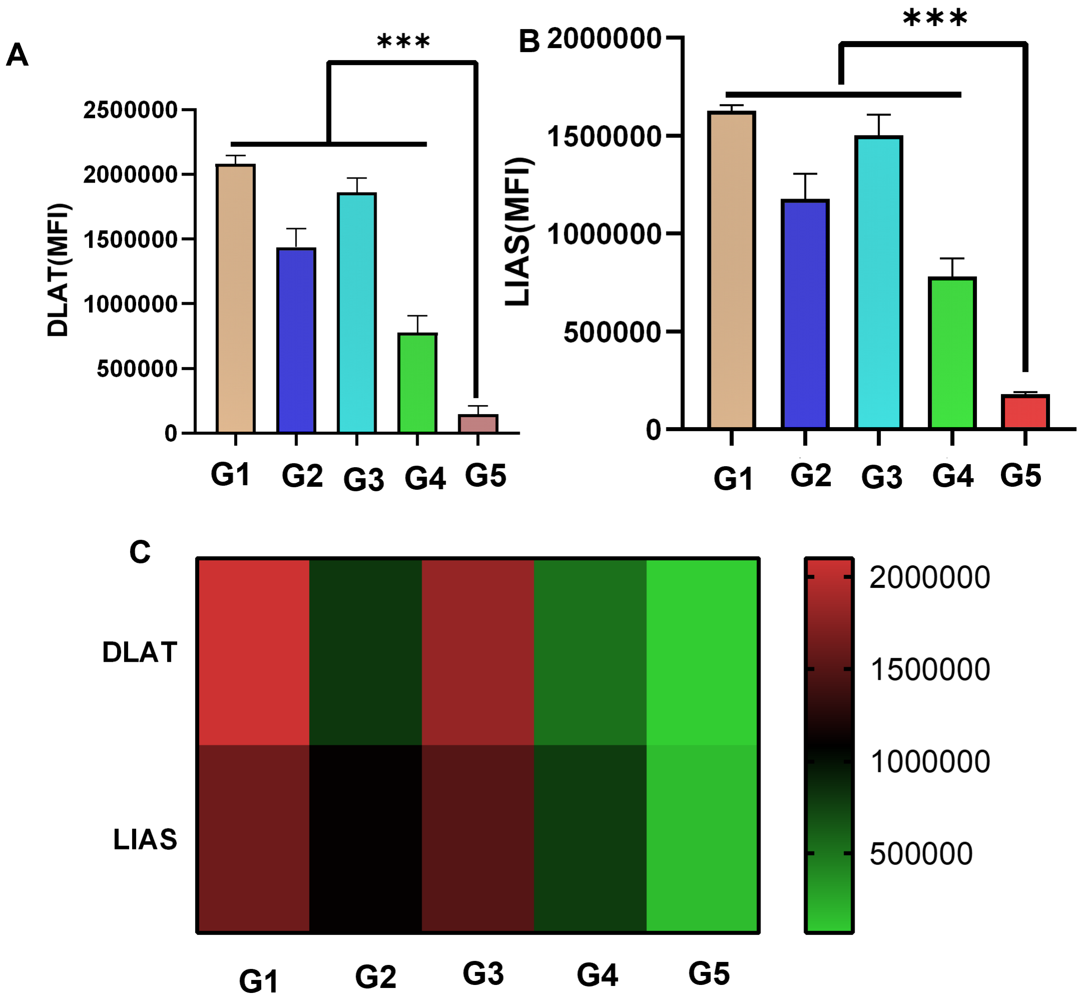


**Figure S15**. Statistical chart of DLAT and LIAS levels in tumor tissues after treatment in different groups. (G1: Sodium alginate, G2: DSF, G3: CuO NPs, G4: DSF@CuO, G5: DSF@CuO Gel). Data are presented as mean ± SD and are representative of three independent experiments. ***p < 0.001.

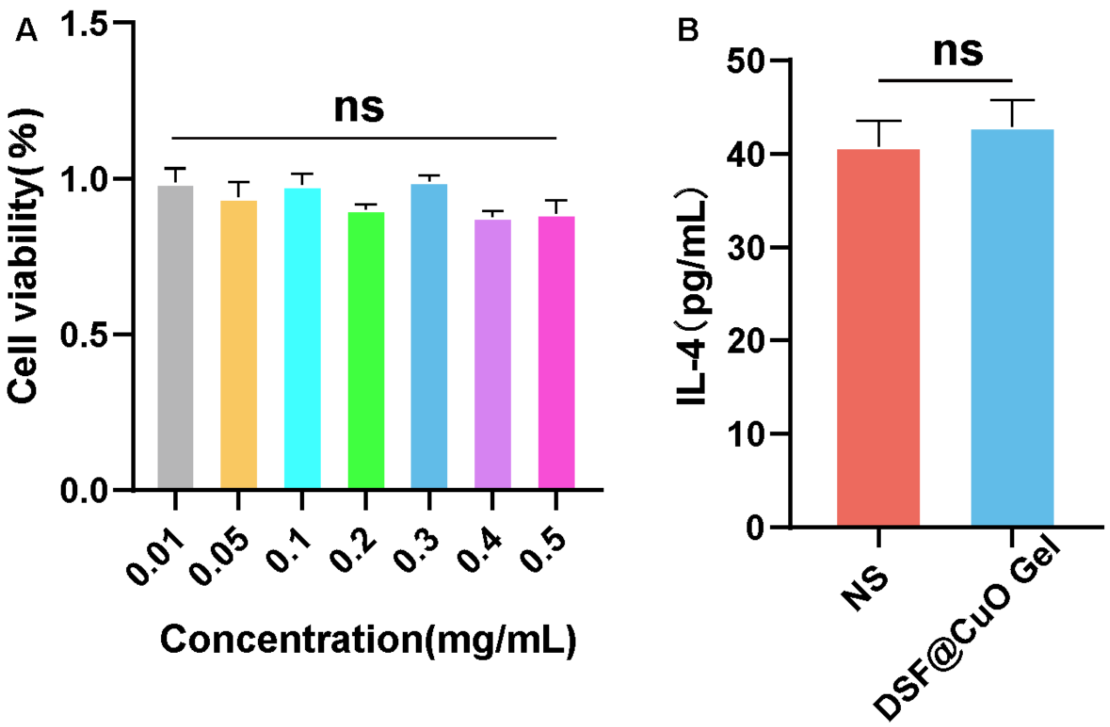


**Figure S16.** Foreign body assessment in different treatment groups. A) The effect of different treatment groups on the survival rate of HUVEs cells. B) ELASA detection of IL-4 levels in mice tumor tissues after different treatment. Data are presented as mean ± SD and are representative of three independent experiments. ns, not significant.

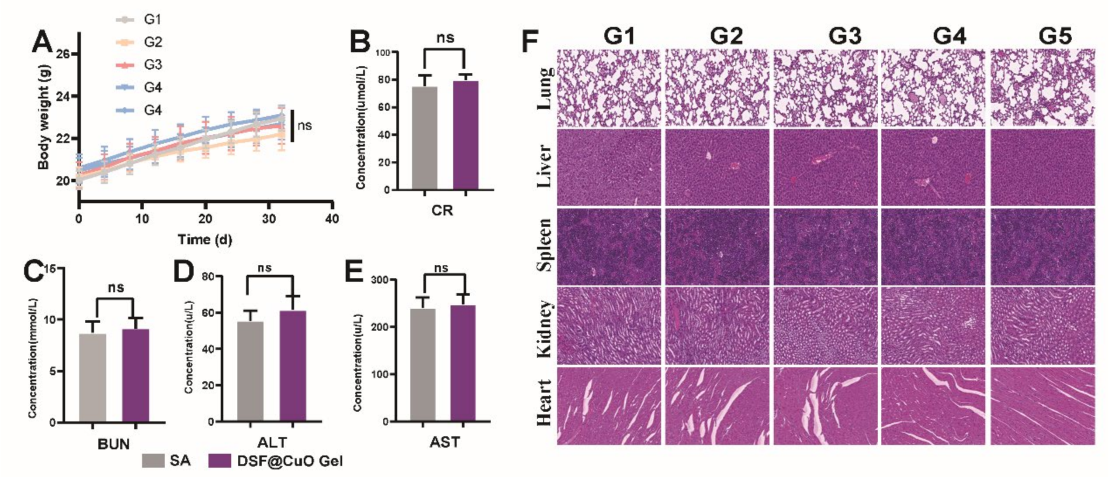


**Figure S17.** Biological safety assessment of DSF@CuO Gel. A) Changes in body weight of mice across different treatment groups (n = 3). B-E) Biochemical analysis of the serum of mice with various treatments (n = 3). F) H&E staining images of important organs from mice after completion of treatment across different treatment groups. Data are presented as mean ± SD and are representative of three independent experiments. ns, not significant.
